# Taiwan’s Road to an Asylum Law: Who, When, How, and Why Not Yet?

**DOI:** 10.1007/s12142-021-00644-y

**Published:** 2022-01-10

**Authors:** Kristina Kironska

**Affiliations:** Department of Asian Studies, Palacky University in Olomouc, Olomouc, Czechia

**Keywords:** Asylum law, Refugees, Taiwan

## Abstract

Taiwan is considered to be one of the most progressive countries in Asia but has no asylum law. Does it need one? Many in Taiwan, including officials and politicians, claim that the regulations that are currently in place are sufficient. There are, however, some people in Taiwan who require protection, and the government is not able to respond effectively in the absence of an asylum law. The author has identified several different groups in Taiwan that would benefit from an asylum law — from Hong Kong protesters facing persecution, through Chinese dissidents or descendants of the ROC army from the Thai-Myanmar border region, to Turkish people with revoked passports; grouped into two major categories — persons from the PRC, Tibet, Hong Kong, and Macau (group 1) and persons from other countries (group 2). The draft of the asylum law has been sitting in the Parliament for 14 years, and the reason for it not yet having passed is the “China Factor.” The Taiwan-China relationship thus cannot be disconnected from this issue, and the article discusses the three most common concerns with regard to this in the Taiwanese society. While these are legitimate concerns, they could be solved by adopting a dual asylum system dealing with group 1 and group 2 separately. Compared to UN member countries, Taiwan is on its own when it comes to the asylum issue, although adopting an asylum law is part of a broader push to bring Taiwan’s legal system in line with international human rights law. The article provides a comprehensive description and analysis of the refugee situation in Taiwan; it is based on document studies and interviews conducted in Taipei in autumn 2020.

## Introduction

In response to persecution, poverty, unemployment, and climate change, hundreds of thousands of people are moving; when legal avenues of migration are denied, people resort to alternative methods. It is a transnational issue that concerns all countries and territories, but Taiwan^1^ has so far stayed out of it. This raises a number of questions. *Can Taiwan really avoid getting involved? Does Taiwan receive any people seeking asylum? In the absence of a refugee-centered law, is Taiwan able to respond concretely and effectively to the related issues in its direct vicinity and at home?*

Due to the political status of Taiwan, the challenges it faces are quite different from other countries. Because the ROC was replaced in the UN China seat by the PRC, Taiwan has  not signed up to UN treaties on the handling of asylum seekers and refugees (see the Appendix). For the purposes of the present article and according to the UN Refugee Agency (UNHCR), an asylum seeker is someone who is seeking international protection but whose claim for refugee status has not yet been determined. The term “refugee” applies to any person who is unable or unwilling to return to their country of origin owing to a well-founded fear of being persecuted for reasons of race, religion, nationality, membership of a particular social group, or political opinion.

Although debates on the issue occasionally occurred for more than 10 years, there has been no progress on the draft asylum law since its second reading in July 2016. One significant point of contention is to what extent an asylum law should address not only people from “uncontroversially” foreign countries, such as the Rohingya in Bangladesh, but also people from China, Hong Kong, and Macau. As with any issue that touches on cross-strait relations, the situation is complicated: On the one hand, the government celebrates Taiwan’s status as a beacon of human rights; on the other, extending asylum to PRC citizens risks stoking tensions with Beijing. The deteriorating human rights situation in Hong Kong provides yet another wrinkle to the debate.

Although not a UN member (thus not able to become party to most international human rights treaties), Taiwan has accepted five international human rights treaties, among them in 2009 the *International Covenant on Civil and Political Rights*^2^ (ICCPR) and the *International Covenant on Economic, Social, and Cultural Rights*^3^ (ICESCR). Although the UN Secretary-General rejected Taiwan’s request to deposit the instruments of ratification, the human rights protection provisions in the two covenants (including the *non-refoulment* obligations) have domestic legal status in Taiwan (Chen and Cohen 2018).

Since Taiwan cannot participate in the reviews undertaken by the UN Committees mandated to monitor states’ compliance with the two covenants, it periodically invites independent human rights experts to review the country’s progress. The first review in 2013 recommended, among others, setting up refugee protection and political asylum mechanisms in order to comply with the *non-refoulement* obligations under Articles 6 and 7 of the ICCPR (Chiu 2014). Moreover, the principle of *non-refoulement* is generally regarded as part of customary international law (although with a lack of consensus as to its status — *jus cogens* or not), and thus binding even for states that are not parties to any of the treaties previously mentioned (Rights in Exile Programme n.d.). The second review in 2017 reconfirmed the importance of the implementation of international human rights law and standards in Taiwan. The Review Committee reiterated the previous recommendation of a speedy adoption of an asylum law and in relation to the non-refoulment principle reminded the Taiwanese government of “*the fact that Article 7 ICCPR already provides an absolute prohibition to extradite, expel or return any person to another country or jurisdiction where he or she would face a serious risk of being subjected to torture or other forms of ill treatment, including capital punishment*” (International Review Committee 2017). The third review is coming up in 2022 (postponed due to the COVID-19 pandemic), but many of the findings from previous years are still the same. Taiwan should thus elevate the standard of human rights to match the world standard. By doing so, it would also reinforce the idea that human rights are universal and demonstrate that Taiwan can be a global leader in the protection of such rights.

The main aim of this article is to demonstrate that Taiwan cannot ignore the refugee issue, because there are various groups of people that require protection and the government is not able to respond effectively in the absence of an asylum law. The situation in Hong Kong highlights this need, but the Hong Kong protesters are not the only group of people in need of protection. The author has identified several different groups, some are Overseas Chinese, others are compatriots (as defined by the Constitution, which claims the ROC to be the legitimate government of China) or regular foreigners; they are grouped into two categories — (1) persons from the PRC, Tibet, Hong Kong, and Macau and (2) persons from other countries. This article provides the first comprehensive description and analysis of the refugee situation in Taiwan in English (although many good articles about partial issues exist in Chinese).

A qualitative research based on document studies (English and Chinese) and individual interviews was conducted in Taipei in autumn 2020. Interviewed were Taiwanese NGO workers, scholars, journalists, and other experts in the field, as well as one legislator who was part of the law drafting process. Full consent was obtained from the participants prior to the study and they all obtained the first draft of this paper for review. They are referenced throughout the paper. Knowledge generated from the content analysis of the interviews is based on participants’ unique perspectives and grounded in the actual data. Such a method allows for new insights to emerge as described by Kondracki and Wellman (Kondracki and Wellman 2002).

As with the majority of studies, the design of the current study is subject to limitations. The first is not having interviewed, with one exception (a Turkish scholar living in Taiwan), people concerned — in this case, participants of the above-mentioned groups that could benefit from Taiwan having an asylum law. The second limitation concerns the limited access to Taiwanese political circles involved in the drafting.of the law.

The article is organized as follows: It first explores the current situation with reference to past events; afterwards, it identifies two groups of people (with different legislation applying to them) that would benefit from an asylum law in Taiwan; followed by a discussion of the reasons preventing the adoption of a comprehensive law (the so-called “China-factor”); and the last part provides a conclusion with some concrete suggestions.

## Current Policy and Practice in Taiwan

Illegal migrants usually arrive in Taiwan on tourist, commercial, transit, or entry visa and then overstay. Only a small number of people enter Taiwan using other pathways, such as fishing boats. Very often they are smuggled into Taiwan to work, and every year, undocumented people are apprehended onboard of Taiwanese fishing boats in the waters off the coast. One of the biggest such operations occurred in 2017, when forty undocumented Vietnamese were intercepted on a Taiwanese fishing boat in waters off New Taipei City (Liao 2020).

Taiwan receives asylum seekers (both self-identified and those already recognized by the UNHCR) every year, and the authorities are unclear regarding how these cases are to be processed, with each authority claiming it is not their responsibility. Immigration only considers how to grant foreigners the right to stay in a country, but refugees require much more than that. Currently, applications are handled on a case-by-case basis with very different outcomes. With such a system, the majority of asylum seeker cases have relied on civil society groups (such as the Taiwan Association for Human Rights, TAHR), which have had to rely on their own resources to provide accommodation, medical treatment, or psychological counseling (Chou and Chiu 2019). While some have managed to receive some sort of support (such as visa extensions or negotiations with third countries) and stay temporarily in Taiwan, others have been deported without taking into consideration the risk of harm if they are returned to their place of origin (despite the *non-refoulment* obligation, which is binding through the domestic adoption of the ICCPR).

This does not mean Taiwan has never accepted anyone. A few examples from the past include in the early 1960s, during large-scale ethnic tensions between Indonesian Chinese and indigenous people in Indonesia, when Taiwan sent two warships to receive those ethnic Chinese who wanted to leave (Wang 2011); admitting some 6,000 Vietnamese refugees during the 14 years from 1976 to 1990 and placing them in refugee camps on Penghu islands (at the time, the camps were still under martial law, so they were managed by the army) in the Rende Project (Chen 2017); and accepting more than 2,000 refugees from the Indochina region floating in the South China Sea in the Haipiao Project (Lee 2017). Many of the people Taiwan accepted under these projects were Overseas Chinese and were eventually allowed to settle in the country.

Political rivalry in the past between the PRC and ROC led to the creation of the specific immigrant category for Overseas Chinese, based on the patriarchal^4^*jus sanguinis* principle which remains in place to this day (Tang 1995). Overseas Chinese who can prove that they or their parents have at one point held an ROC passport can register for the Overseas Chinese Certificate and with that apply for settlement in Taiwan — between 1982 and 2007, a total of 230,999 such people settled in the ROC (Wang 2011).

Taiwan is active also with regard to providing financial help to refugees abroad. The country is part of many development projects around the world aimed at meeting the urgent needs of those impacted by regional turmoil. For example, Taiwan has provided over US$20 million in humanitarian assistance programs since the Syrian civil war broke out in 2011 (Taiwan Today 2020). There is also an agreement with Australia to provide medical treatment for refugees and asylum seekers in Nauru (bringing them to Taiwan for surgery if necessary); the MoU is, however, confidential, so there is not much known about the content of the agreement or the numbers of people flown to Taiwan (CHIU, personal communication, October 2020).

In the past, there were a few attempts to enact an asylum law. A draft law (inclusive of Chinese nationals) was first sent to the Parliament in 2005 with the aim “*to safeguard the status of refugees, to protect the rights and benefits of refugees, and to encourage international cooperation on matters related to human rights*” (TAHR 2012), but it has not been discussed any further. Similarly, in 2011 and 2012, draft laws were submitted, discussed in public hearings, but never picked up by any parliamentary committee for further discussion. The Minister of Interior objected that there were not enough experts to handle this issue and Taiwan needed more supplementary laws to facilitate an asylum law. At the end of the parliamentary term (under the Nationalist Party, KMT) in January 2016, all draft laws, including the draft asylum law, were removed and erased.

In 2016, with the start of the new parliamentary term (under the Democratic Progress Party, DPP), four versions of the draft law were submitted by (1) Yu Mei-Nu (DPP) and Apollo Chen (KMT), (2) Hsiao Bi-khim (DPP, now *de facto* ambassador in the United States), (3) Tsai Yi-yu (DPP), and (4) the Executive Yuan. All of these draft versions excluded Chinese nationals, as by that time there was a consensus that it would be easier (politically) to pass such a version of an asylum law, and PRC nationals would be dealt with later (discussed later in the article). The draft passed the first reading under the Committee of Internal Administration but has subsequently not been sent through to second and third readings.

**Timeline of Taiwan’s Asylum Law Draft**

**Table:** 

2002	The Research and Development Evaluation Commission of the Executive Yuan (under the DPP) adopted a resolution about establishing an asylum system.
2003	The Executive Yuan (DPP) sent a letter to the Mainland Affairs Committee of the Ministry of the Interior for further research and review of the asylum law.
2005	TAHR submitted a draft asylum law to the Legislative Yuan. It has not been discussed.
2006	The Executive Yuan (DPP) convened the Ministry of Interior, the MOFA, the MAC, and other relevant departments to discuss the issue of refugees coming from Mainland China, Hong Kong, and Macao.
2007	The Executive Yuan (DPP) sent the draft asylum law (including provisions regarding refugees from China) to the Legislative Yuan for review. There was no substantive discussion.
2009	The Minister of the Interior requested the Executive Yuan (KMT) to withdraw the draft from the Legislative Yuan, and launched a new one with Article 15 deleted: “*The asylum law shall also apply to the people of the Mainland and residents of Hong Kong and Macao.*” The NIA reviewed the new draft and proposed “not promoting” it.
2011	The DPP (as a party) submitted a draft asylum law to the Legislative Yuan (under the KMT). It has not been discussed.
2012	Another draft asylum law (TAHR’s version) was submitted, this time proposed by individuals from both the DPP and the KMT. The lead person for the KMT was Apollo Chen, and for the DPP Hsiao Bi-khim (now *de facto* ambassador to the United States).
2012-2016	The draft law was discussed (public hearing) but never picked up by any committee for further discussion. The Minister of Interior objected that there were not enough experts to handle this issue and Taiwan needed more supplementary laws to facilitate the asylum law. At the end of the parliamentary term (under the KMT) in January 2016, all draft laws, including the draft asylum law, were removed/erased.
2016	In 2016, after the start of the new parliamentary term (under the DPP), the Legislative Yuan reviewed the draft asylum law again. There were four versions of the draft law by: 1. Yu Mei-Nu (DPP) and Apollo Chen (KMT) 2. Hsiao Bi-khim (DPP, now *de facto* ambassador in the United States) 3. Tsai Yi-yu (DPP) 4. Executive Yuan All versions were similar — excluding people from the PRC — and were discussed on the floor simultaneously (under supervision of the Committee of Internal Administration). The draft law passed the first reading but was not put into the second or third readings.

Source: Compiled by the author

According to former legislator Yu Mei-nu (personal communication, December 2020), the most discussed issues were (1) Whether people persecuted because of their sexual orientation or gender identity should be included (part of the first version of the draft); (2) how to define a refugee, review the status of an asylum seeker, and grant asylum or not (the government proposed a primary review which would automatically exclude criminals, people who have been granted asylum elsewhere or people who already applied and have been rejected; civil groups opposed this, claiming this process alone would eliminate most cases); (3) whether or not to automatically reject (as proposed in the fourth version of the draft) asylum seekers who pass through another country (even if it is just changing flights, which is almost inevitable due to Taiwan’s location) that has the ability to grant asylum (reference taken from Canada and Japan); and (4) what the next steps would be if an asylum seeker were rejected (civil groups wanted specific regulations to be included in the law, while the government preferred more flexibility; the committee went forward with the government’s suggestion but consultations with civil groups would be mandatory). There were many other political considerations, such as what department would be in charge and how it would proceed. If the law was passed, the National Immigration Agency (NIA) would not be able to coordinate with other departments, as only the Executive Yuan can do that. Many governmental agencies were hesitant, and there were still many issues without any consensus as to how to proceed, and so the draft law did not pass to the next reading.

When the parliamentary term ended in January 2020, all the discussed laws were erased, meaning that a new draft law would have to be submitted and discussed again. Currently, there is no political will to do so. There are still many questions lingering over the definition of a refugee and limits on numbers, as well as their rights and obligations. Moreover, officials and politicians, even President Tsai Ing-wen and Premier Su Tseng-chang, claim there is no need for an asylum law because the regulations that are in place are sufficient (Aspinwall 2019). However, adopting an asylum law is part of a broader push to bring Taiwan’s legal system in line with international human rights law.

## People in Need of Protection

Due to Taiwan’s political history with the PRC, it has a very complicated and outdated immigration system and foreigners — meaning people living outside of Taiwan — are categorized into six groups: Chinese from the PRC; Overseas Chinese with an ROC passport; Overseas Chinese without a PRC or ROC passport; Taiwanese who have been naturalized in other countries; PRC Chinese who have been naturalized in other countries; and other foreigners^5^ (Wang 2011). This division has created a very specific situation, in which different groups of foreigners in need of protection face legally very different situations and different legislation applies to them. Namely, there have been identified several groups of people that would currently benefit from an asylum law in Taiwan, grouped for convenience into two groups — persons from the PRC, Tibet, Hong Kong, and Macau and persons from other countries.

### Group 1: Persons from the PRC, Tibet, Hong Kong, and Macau

#### Hong Kong Protesters Facing Persecution

People from Hong Kong are currently the most discussed group when it comes to the topic of asylum and refugees in Taiwan. In 2019 and 2020, numerous rallies in support of Hong Kong took place in the streets of Taipei. A 2020-survey by Academia Sinica shows that the majority of Taiwanese people support Hong Kong, and young people have a particularly strong inclination to support the cause (Institute of Sociology as 2020). Moreover, the Hong Kong community has a good image in Taiwan, since many famous people, including the poet Albert Leung or political scientist Simon Shen, decided to settle there.

The Taiwan government has also expressed its support for the Hong Kong protest movement, but it initially also said that the legislative review process for the draft asylum law is incomplete, and thus Taiwan could not receive people who are forced to flee in an emergency — they would be handled on a case-by-case basis. Factors, such as “political considerations,” play a big role in permitting political refugees from Hong Kong or Macau to stay in Taiwan. Taiwan applies a different policy towards the people of the Mainland and the two SARs — they are treated neither as foreigners nor as citizens. The approach towards Hongkongers is defined by the Laws and Regulations Regarding Hong Kong and Macao Affairs (LRRHKMC), while Mainlanders are dealt with under the Act Governing Relations between the People of the Taiwan Area and the Mainland Area (AGRPTAMA).

Officials in Taiwan, including one of the core legislators, Cheng Yun-Peng (DPP), claim that there is an adequate legal framework to process Hong Kong asylum seekers who participated in the anti-extradition bill protests in 2019 and the National Security Law protests in 2020 (CHIANG, personal communication, November 2020). Although Article 18 of the above-mentioned LRRHKMC law states that “*necessary assistance shall be provided to Hong Kong or Macau residents whose safety and liberty are immediately threatened for political reasons*” (Mainland Affairs CounciL 2017). It does not specify in any further detail on how this should be done or by whom.

The Mainland Affairs Council (MAC) under the Executive Yuan focuses mainly on China and the Chinese; Hong Kong is only a small part of their agenda, and the refugee issue is not at all. And even though some MAC civil servants are willing to help, other governmental departments, such as the Ministry of Labor or Education, do not feel any responsibility, which would not be the case if Taiwan had binding legislation in this regard.

Most people from Hong Kong are encouraged to seek other pathways to receive residency in Taiwan — these include working in Taiwan, investing in a start-up to obtain an entrepreneur’s visa, or studying to obtain a student visa. This was the case of the former owner of Causeway Bay Books Lam Wing-kee, who was supposedly abducted in 2015 by Chinese agents for selling books banned in China. He came to Taiwan, crowdsourced money, and revived his business in Taipei — this way getting a permit to stay, rather than applying for asylum. The only help the Taiwanese government provided was extending his visitor’s visa, giving him more time to make arrangements.

This approach has a major flaw: only white-collar workers with a monthly salary of more than 45,000 NTD or those with an investment of six million NTD have the “right” to immigrate to Taiwan (Hong 2020). In practical terms, this means that only professionals and rich people can take advantage of this channel, while most of the protesters in Hong Kong are students and workers. These people thus have to rely on the recently created network of NGOs comprised by the TAHR, Economic Democracy Union, Taiwan Citizen Front, and other organizations (not willing to disclose their identity) committed to helping Hongkongers wanting to settle in Taiwan. For example, if there is a person who wants to pursue tertiary studies in Taiwan (as an alternative pathway to asylum), but he or she is below eighteen and without a high school graduation certificate, they have to enroll in a Taiwanese high school to get the certificate first — which is not possible without a residence permit (and without a residence permit, public schools will not accept foreign students). Another possible problem is when a Hongkonger’s passport expires — the person is then stuck in Taiwan. And there are many other situations that need special consideration. The organizations in the above-mentioned network cooperate and negotiate with the Taiwanese government in order to help solve such precarious situations — they introduce private schools that do not require resident permits to enroll, raise funds to pay for tuition, help find accommodation, jobs, etc. According to the New York Times (2019), there were around 200 such cases in 2019 (the NGOs are not willing to disclose the number).

According to Min-yen Chiang (personal communication, November 2020) from the Economic Democracy Union, the main goal of the NGO network is to legalize the stays of Hong Kong protesters — in the short term by amending the LRRHKMC (most notably the above-mentioned Article 18, which is the most relevant to political asylum, but in its current form way too vague) and in the long-term by adopting an all-encompassing asylum law.

The prospects are, however, bleak. In May 2020, when China announced the National Security Law for Hong Kong (which grants the PRC broad powers to crack down on a variety of so-called political crimes), in Taiwan the Head of MAC responded that they were preparing to amend the existing regulation; however, this was later refuted by the Head of the Legislative Yuan, Yu Shyi-kun, who said amendments were not necessary and Taiwan was fully prepared.

In June 2020, MAC announced that a new Taiwan–Hong Kong Service and Exchange Office would be set up to come up with a rescue plan for Hong Kong. The office would be funded by the government and cooperate with human rights and civil society groups to help with residency, settlement, employment, and protection issues (Huang 2020). The new office is not open to the public and, according to the NGO network, it has not been tested yet, since the pandemic has halted many arrivals from Hong Kong.

The civil society in Taiwan hopes for an inclusive approach — a kind of an SOP as to how to take care of the incoming Hongkongers (including all aspects of life, such as accommodation, medical needs, education, insurance, and psychological help), not just accepting them to move to Taiwan (YU, personal communication, December 2020). In the long run, this could push the asylum law issue in Taiwan — after establishing a practice of accepting people from Hong Kong, the old law drafts could be revisited and enacted.

#### Chinese Dissidents

Perhaps the most controversial group of people from a political perspective are Chinese from the PRC. The control over them in Taiwan is much stricter than that over other foreigners for reasons of national security, and the issue of Chinese refugees is a particularly sensitive one.

From the 1950s through the 1970s, communication and cooperation across the Taiwan Strait were strictly prohibited. After lifting martial law, the policy towards the PRC began to relax, and in 1991, Taiwan even set up the Straits Exchange Foundation (supervised by the MAC), a government-funded non-profit organization in charge of negotiating and implementing agreements with its counterpart in China (Chen and Cohen 2018).

In the 1980s, numerous Mainland Chinese risked reaching Taiwan illegally via sea, most hoping to earn a better living on the island. If detected, the vessels would be forced to turn back. In 1990, dozens of Chinese people on these boats were caught and locked below deck by the Taiwanese authorities to prevent escape and were later found dead (Kristof 1990). Public outrage prompted the negotiation of the Kinmen Agreement, which is still in effect today and covers the safe repatriation of “residents who entered the other side in violation of relevant rules” and “criminal suspects and criminals.” The number of returned people peaked at more than 5,000 a year in the 1990s and gradually declined to fewer than 100 in recent years as China’s economy has been developing fast and the economic incentives for going to Taiwan have diminished (Chen and Cohen 2018). However, because of the language and cultural similarity, Chinese dissidents still choose Taiwan as their refuge.

Most people escape from China by boat or swim to Kinmen. The first such Chinese dissident seeking political asylum arrived in 2002 (Carey n.d.). Once in Taiwan, these people are sent to one of the detention centers (in Taiwan the detention of unauthorized arrivals is mandatory), since there are no refugee shelters.

Since there is no asylum law in Taiwan, all these cases are solved by a special project with the help of human rights organizations. The solution usually involves the Ministry of Foreign Affairs (MOFA), the MAC, the NIA (set up in 2005), and several laws, such as the Immigration Act, the AGRPTAMA (most notably Article 17, which vaguely refers to political and other considerations for allowing permanent stay in Taiwan), and the Rules Governing Permits for People from Mainland China Setting Up Dependent and Permanent Residence.

Dissident Wang Jui came to Taiwan in 2014 and attempted to sail a boat to the United States with four other Chinese dissidents. The attempt failed, and all but Wang were repatriated to China. He was held in detention for 4 years before being sent to the United States (Tang 2018). This is one of the rare cases where a Chinese dissident managed to get from Taiwan to the United States in pursuit of political asylum. Many others are either repatriated or remain in Taiwan with a temporary visa (not allowing them to work) and a modest financial subsidy by the MAC (Tang 2018). Only very few manage to get a long-term residency permit in Taiwan. In 2014, for example, nine Chinese nationals received this status (Hsiao 2014).

A more recent case made headlines in Taiwan, when two Chinese asylum seekers registered at UNHCR in Thailand were stuck at Taiwan Taoyuan International Airport. They left Thailand because of safety concerns flying from Bangkok to Beijing via Taipei, where they did not get on the outbound flight and instead requested asylum on the grounds of political persecution in China (Taipei Times 2019). The Taiwanese officials were quite stuck about what to do, since they knew it would be difficult for them to get help from the UNHCR (since Taiwan is not a UN member state). The Refugee agency is, however, allowed to cooperate with Taiwanese NGOs, and TAHR managed to verify the identity of the two Chinese men in question. Based on this, the Chinese men found a guarantor organization that would vouch for their stay in Taiwan, and after 4 months, they were finally let into Taiwan (from the airport) on the basis of “professional exchanges” until they were granted asylum in a third country (CHIU, personal communication, October 2020).

Why are some Chinese dissidents able to remain while others are repatriated? Since these cases are dealt with on a case-by-case basis, it usually depends on the dexterity of the NGOs and government agencies involved. According to the above-mentioned Article 17 of the AGRPTAMA: “*The Ministry of the Interior may permit specifically on a case-by-case basis any of the people of the Mainland Area to have a long-term residency in the Taiwan Area out of political, economic, social, educational, science-tech or cultural consideration and may restrict the categories and quota for residency applications*” (Mainland Affairs Council 2019).

#### Exiled Tibetans

Tibetans in Taiwan are in an ambiguous position — they are neither citizens nor foreigners; they are not treated as compatriots nor are they viewed as refugees. Although symbolic rather than substantial, the ROC used to claim its sovereignty over Tibet (when the ROC was founded in 1911, the KMT government explicitly claimed its sovereignty over this territory), despite losing this sovereignty in 1949 to the Communists. Tibet was invaded and subsequently occupied by the Chinese Communist Party in what is called the 1959 Lhasa Incident. As a result, the 14th Dalai Lama and thousands of his followers escaped and became refugees or stateless people in India.^6^ In Taiwan, Chiang Kai-shek declared support for the Tibetans’ anti-Communist stance but did not provide any real assistance. The government simply regarded the exiled Tibetans in India as “overseas Chinese from Tibet.”

Tibetans came to Taiwan mainly from India and Nepal during the Cold War. A few dozen belonging to the Tibetan elite, who had special relations with those at the top of the KMT, settled in Taiwan and their children adapted well. Others came for a training program that involved working in a factory or for the education program for Tibetan children. Many factory workers escaped and overstayed their visa permits. Tibetan children receiving education in Taiwan got accustomed to life on the island and were not willing to leave, and these now constitute the population of “Tibetans in Taiwan” (Pan 2015). Although they came through official channels, they became illegal immigrants in Taiwan, and the government should address their situation. In the past, there was the ministry-level Mongolian and Tibetan Affairs Commission (MTAC), but it was disbanded in 2017.^7^ Today, there is no special office for Tibetan affairs in Taiwan, and so such people have to rely on NGOs, such as TAHR or Taiwan Friends of Tibet for help.

There are also those who arrive illegally. It is difficult for Tibetans to acquire valid travel documents in China, India, or Nepal, and so they often decide to travel with fake passports. For example, in 2008, 134 Tibetans in exile arrived in Taiwan with false Nepali or Indian passports (Lin 2018). They require the same treatment as those who came to Taiwan by legal means. Some others claim to be Tibetans, but the Taiwanese government has doubts about their identity and suspects them to be Nepalis (PAN, personal communication, November 2020).

Currently, there are about 2,000 Tibetans in Taiwan (Carey n.d.), including many Buddhist monks who come to Taiwan’s Buddhist centers to teach (they need to leave every 6 months to renew their stay permits), people who came through official MTAC channels, and also those who arrive illegally.

The Immigration Act (Article 16) was amended a few times to allow Tibetans to apply for residence, although it only applies to people who entered Taiwan before June 2016 and the standards are too high and difficult to meet (an applicant must earn at least double the Taiwanese average monthly salary; own property worth more than 5 million NTD; possess a specific professional or technical skill; or serve as a skilled employee in the high-tech industry) (Pan 2015). Some ask for exceptions, and their cases are dealt with on a case-by-case approach.

Although exiled Tibetans with Indian identity papers can immigrate and travel internationally, the Taiwanese government regards them as stateless, and according to Taiwanese law (Regulations Governing Visiting, Residency, and Permanent Residency of Aliens), “*stateless person who entered Taiwan with a visitor visa may not apply for residency*” (Ministry of the Interior 2018). Even exiled Tibetans married to Taiwanese are denied the rights that most foreign spouses enjoy (access to healthcare and the ability to work legally), they have to leave the country every 2 to 6 months and re-apply for a dependent spouse visa at the Taipei Economic and Cultural Office in India. Only very few Tibetans, with the help of local human rights organizations and official legislators, have received Taiwanese ID cards (Pan 2015).

The residency of Tibetans remains an unsolved issue in Taiwan. Some are running away from India and Nepal, where they are deprived of the rights to education, work, or holding property; others flee China’s tightening grip on religion. Many of those fleeing from China are nuns or lamas who make a detour to Nepal before coming to Taiwan (Huang 2020). However, once they are repatriated to Nepal, it is very likely they will be sent to China, where they will encounter cruel treatment and not receive fair trials.

#### Descendants of the ROC Army from Thai-Myanmar Border Region

During 1960–1961, the so-called Sino-Burmese border Surveying and Security Operation occurred — a series of battles between the Chinese Nationalists of the ROC and the Chinese Communists of the PRC around the China-Myanmar border and in Northern Thailand. Some soldiers of the ROC army and their descendants were left behind, and the governments of Myanmar and Thailand refused to grant these people citizenship, so they became stateless.

The Ministry of Education in Taiwan recruited these stateless Thai and Myanmar students in order to promote the Chinese language, but they are supposed to leave Taiwan after graduation. TAHR, Judicial Reform Foundation, Legal Aid Foundation, and Thai-Myanmar Chinese Refugee Rights Association are all fighting for a change in the Immigration Act so that these students can be allowed to stay in Taiwan and even get citizenship. So far, approximately 1,000 Thai and Myanmar students have received Overseas Chinese Residence Visas in Taiwan, and around 200 students were permitted to naturalize in Taiwan (Carey n.d.).

Around 250 overseas Chinese from Myanmar and Thailand come to Taiwan to study each year using forged passports (Taiwan Today 2008). Once these students complete their studies, their only options are to stay illegally in Taiwan or return to a stateless existence in their host country.

On the day when the Immigration Act was promulgated, 21 May 1999, the Taiwanese government declared an amnesty for stateless overseas Chinese who had entered the country before that date. However, a few more thousand descendants of former KMT soldiers have entered the country since then and are living in the country illegally (Taiwan Today 2008). In 2016, Article 16 of the Act was amended, but only allowing those to stay “*who have been allowed to enter the country for the purposes of attending school or receiving technical training by the Ministry of Education or the Overseas Compatriot Affairs Commission between 21st May 1999 and 31st December 2008*” (Ministry of the Interior 2016).

### Group 2: Persons from Other Countries

Over the past decade, Taiwan has received asylum seekers from Syria, Turkey, China, and Tibetans in exile, but also from other countries not yet mentioned, such as Uganda, South Korea, or Egypt, who came to seek protection for one reason or another. There are, however, no government statistics about asylum seekers, and TAHR only finds out about them from the media or its contacts in the government — it counted forty such cases between 2013 and 2017 (see Table 1).

**Table 1:** Asylum seekers in Taiwan between 2013 and 2017

Country of origin	No. of women	No. of men
Columbia	1 transgender person	
Uganda	1	1
Gambia	-	2
Egypt	-	1
Turkey	-	3
Hong Kong	1	-
Tibet	5	13
China	1	10
South Korea	-	1

Source: Taiwan Association for Human Rights

Some of those that come to Taiwan to seek protection are LGBTI people, who face gender discrimination or even severe punishment for homosexual activity in their home countries (for example Uganda) and have heard Taiwan is an LGBTI-friendly country. Some are seeking protection for other reasons. Then there are also the naturalized citizens (that have to give up their original nationality) whose Taiwanese citizenship can be revoked by the government for certain illegal activities (until 2015, contracting HIV was one of them), which would leave them effectively stateless.

In 2003, there was a North Korean woman who arrived on a cargo ship to Kaohsiung. She escaped from China, where she jumped into the sea and was rescued by a Panama-registered cargo ship sailing to Taiwan (Sydney Morning Herald 2003). Instead of being regarded as an illegal foreigner and following the usual Taiwanese pathway of sentencing and then deporting people who entered Taiwan illegally, officials contacted the South Korean Representative Office in Taipei, which was then able to negotiate with the Taiwanese MOFA for the woman to be allowed in Taiwan as a transit station on her way to South Korea, where she could seek political asylum (MOFA 2003).

Some people escape from war-torn areas and enter Taiwan on fake passports in an attempt to transfer to a third country where they can apply for asylum and/or reunite with their spouses that are already there and have obtained refugee status. According to the UNHCR, “*refugees and other persons in need of international protection who have no other country than the country of asylum or resettlement to lead a normal family life together should be entitled to family reunion in the country of asylum or resettlement*” (Annual Tripartite Consultations on Resettlement 2001). However, when discovered by the NIA in Taiwan such individuals (including those already recognized as refugees by UNHCR elsewhere) are deported. This type of entering is regarded as a crime, although this ignores the important background factors that brought these people to the country. In 2015, three Kurds entered Taiwan in an attempt to transfer to Europe (Chiu 2014). In 2018, some Syrian Kurds took a transit flight to Taiwan (Huang et al. 2020). In 2019, Syrians who escaped their war-torn country arrived in Taiwan *en route* to France to unite with their spouses who had already obtained refugee status there (Lai 2019). There might have been more, but there are no official reports about deportations. All these cases were dismissed by the NIA as illegal immigrants who used fake passports, sent to a foreigner detention center, and prosecuted for forgery (under the Immigration Act and the Criminal Code). The court ordered them to be deported after having served their sentences. These people took a transit flight to Taiwan as asylum seekers, and even if there is no asylum law in Taiwan, the state should at least be bound to the principle of *non-refoulement*, since Taiwan has ratified and incorporated into its domestic legal system the human rights protections of the ICCPR.^8^ By forcibly returning people to their countries of origin despite the risk that they may incur severe human rights violations there, Taiwan fails its obligation under the ICCPR to establish effective review of the removal decision in order to avoid irreparable harm to the individual.

An interesting case concerns some Turkish people living abroad, including in Taiwan, with revoked passports. The failed military coup in Turkey in 2016 triggered a witch hunt, a result of which was more than 90,000 people being arrested as enemies of the state; state officials and academics dismissed and replaced by people appointed by the Erdogan administration; and many more are being investigated, including many people who reside abroad (Turkeypurge n.d.). Turkey has been pressuring other countries to send Turks involved in the Gülen Movement^9^ (which has been labeled as a “terrorist movement” that attempted the coup by the Turkish government) back to Turkey. The group’s exact scope is difficult to define; people and organizations who say they are inspired by Fethullah Gülen, a Sufi thinker and peace activist, operate around the world, often focusing on cross-cultural, business, and education ties with Turkey (Lin and Lin 2014). Many of them who work and study in Taiwan — some are even married to Taiwanese and have lived there for decades — got their passports revoked or confiscated when they applied for a new one once their old passport expired, and the only way to get a new passport is to apply for a new document in Turkey. They were provided a so-called pink passport, a temporary one-way passport to get back to Turkey. However, since they have been blacklisted by the Turkish government for being a member of the Gülen Movement, traveling to Turkey entails a great risk of being imprisoned. Many others — as of March 2020, there are 304 Turks living in Taiwan (Ministry of the Interior 2020) — still have valid passports but worry about what will happen to them when their passports expire, and they will have to renew them. There are several cases of when Gülen activists living in Taiwan have been harassed (for example, at the Turkish Trade Office in Taipei) or via mobile phones with threats from Erdogan’s supporters (Yu 2019). Since 2016, the Turkish diaspora in Taiwan has been shrinking (exact numbers are not available), because many people applied for travel visas to other countries, for example, Australia, and once they got there claimed asylum based on political persecution. One man traveling together with his family back to Turkey on a pink passport bought flights that required changing in the Netherlands, and once they got to Amsterdam, they claimed asylum (CUBUK, personal communication, October 2020). Those Turkish people who want to remain in Taiwan face an impossible situation, as they cannot apply for asylum which would allow them to stay in the country.

What are their other options? One is leaving for another country and claiming asylum there, although many countries are already aware of the issue and stopped issuing travel visas to Turkish nationals residing abroad. Those who hold an APRC can remain legally in Taiwan (it allows 99 years of residence) but can never again travel abroad (because they do not hold a valid passport). Another option would seem, for those who are married to a Taiwanese citizen, to apply for Taiwanese citizenship. However, to apply for naturalization, one is required to give up one’s original citizenship, which means a confirmation needs to be issued by the *de facto* Turkish embassy in Taipei, and according to the Turkish diaspora living in Taiwan, the office does not provide this document and thus hampers the whole naturalization process (WAFR, personal communication, October 2020). In neighboring South Korea and Japan, they solved this issue by stipulating that this is a special case and giving up the Turkish citizenship is not required in order to obtain local citizenship.

Then there are also children of immigrants born in Taiwan that are considered non-citizens and ineligible for benefits if they are not registered at birth. From 2007 until the end of May 2019, more than 9,000 children were born in Taiwan to foreign parent/s, of which more than 700 did not have citizenship (Wang 2019). According to the NIA, there are on average about 200 pregnant migrant workers or those already caring for a child who disappear from their jobs each year due to the asymmetric contractual relationship between agencies and migrant workers (Wang 2019). If an undocumented person bears a child, it is stateless and without legal residency until it is registered at the respective *de facto* embassy in Taiwan.^10^ However, undocumented migrant workers avoid contacting their *de facto* embassies to register their children, because they fear deportation. Some of these children are abandoned by their parent/s and end up in shelters. If the parent/s cannot be found and the child has no birth certificate, it can get Taiwanese citizenship, although it is a lengthy and complicated process *(*Ministry of Health and Welfare 2019). However, there are other cases that have no easy solution. If the child’s mother is known to be a migrant worker but her whereabouts are unknown, the child is stateless and the respective embassy gets involved — the child is sent to the respective country. Not all countries welcome these children; for example, the Indonesian government cooperates but the Philippines and Vietnam question the identity of such children — and so they remain in Taiwan and would benefit from an asylum law (WANG, personal communication, November 2020). There are also some children that were delivered at home and raised by their undocumented parent/s. According to Yi-Fan Feng (personal communication, November 2020), the Executive Secretary of Harmony Home Taiwan, there are about 200 such cases per year. These children are not covered by health insurance and have difficulty accessing education. 11 Before reaching the age of eighteen, children fall under the Child and Juvenile Welfare and Rights Protection Act, but after that, there is no legal protection for these people and they become ghosts in Taiwanese society (Jian 2016).

Due to the absence of an asylum law, the various cases of these people are handled by NGOs that negotiate with the relevant government institutions, such as the NIA. They are taken care of by these organizations but usually never manage to get a residence permit. Some end up leaving voluntarily for other countries where they can apply for asylum (the Colombian person left for New Zealand and obtained asylum there), and a few get married to Taiwanese. Others manage to stay based on an under-the-table agreement with the government that they are treated like ordinary foreigners, but have no residency, and thus cannot legally work (LIN, personal communication, October 2020). TAHR also mentions a catch-22 case, a Ugandan woman who was asked to prove she was employed before she could get a residence permit, but all the employers she has approached require residence papers in order to employ her.

## The China Factor

If there were no people seeking asylum, the government would have no responsibility to act; the reality however looks different, as we have seen in the previous chapter. Those such as the exiled Tibetans or Turkish people with revoked passports actually exist in reality and cannot be ignored, even if they are not easily categorized into the current international order. There are thus several organizations in Taiwan, such as TAHR, AI Taiwan, or the NGO network dealing with Hong Kong protesters risking riot charges, that are calling for reform of the Immigration Act and adoption of an Asylum Law.

The application of a refugee policy also brings with it political issues, because the recognition of asylum seekers as genuine refugees — directly or indirectly — could label their home country (or parts of it) as unsafe and persecutory. While geo-political concerns and the implications of granting asylum for diplomatic relations are common for other countries too, for Taiwan this could turn out to be politically very problematic — especially in regards to China, but also in relation to Taiwan’s few remaining diplomatic allies (which actually have a record of human rights abuses) or the countries included in the Southbound policy (for example in Myanmar, the issue of the genocide of the Rohingya people is never even mentioned by Taiwanese officials out of fear of offending a country Taiwan has been courting in the absence of diplomatic recognition for a long time) (Anonymized).

There have been many discussions on whether people from the PRC, Hong Kong, and Macao should be included in the law or not. In the Taiwanese political context, the country could get away with not including Chinese people in the asylum law. According to TAHR and Amnesty International Taiwan, all people regardless of nationality should be treated equally and these organizations see adopting a law excluding the Chinese as wrong. On the other hand, South Korea does provide an example of one country with two systems for processing asylum-seekers — for ordinary foreigners seeking asylum and North Koreans (who are not really considered refugees and are granted citizenship of the Republic of Korea). Although it is beyond the scope of this article to provide a detailed analysis of the Korean two-system process, the situation is analogous and the lessons that could be learned in Taiwan are tremendous.

In the past, the consensus was that passing a law that excludes Chinese people from the PRC might speed up the process (and the MAC could then still amend the acts related to the Mainland Chinese and Hong Kong and Macao people to ensure the law is also applicable to them), but this never materialized. Moreover, the current asylum law draft excludes not only the Chinese but also many other people who arrive (even if they only change flights there) from a third safe country with an asylum system. Many NGOs thus regard the current draft as useless.

Taiwan’s draft asylum law has been sitting in the Parliament for 14 years, and the main reason is the so-called “China Factor.” The Taiwan-China relationship cannot be ignored, and there are three popular considerations, all legitimate concerns, when discussing an asylum law in Taiwan:

### Political Sensitivity Surrounding the Two Chinas

Most of the refugees seeking asylum in Taiwan are from China, Hong Kong, or Tibet, and according to the ROC Constitution (still valid in Taiwan), which was adopted in 1946 in Nanking, they are technically ROC citizens, not foreigners. Moreover, a refugee must have crossed an international border (according to the definition of a refugee under the 1951 Refugee Convention), and Taiwan officially still claims to be the legitimate government of the State of China including the Mainland and Hong Kong. Under such circumstances, including exiled Tibetans, Hongkongers, and Mainland Chinese in the asylum, the law becomes very tricky, if not impossible. By including them in the law, Taiwan would officially indicate that these people as non-citizens and imply a changed sovereignty claim. This automatically brings up the topic of Taiwanese *de jure* independence that so many are afraid of because of China’s threat to use force if Taiwan proclaims independence (YEW 2020). According to Yu Mei-Nu (personal communication, December 2020), this is a very sensitive issue, and Taiwan needs to be pragmatic and smart about it in round two of the law drafting to avoid conflict with China.

### National Security

The government and a large part of the Taiwanese population do not want an asylum law because they are afraid of Chinese spies (Lev and Hioe 2019). However, spies can get to Taiwan in many ways other than as refugees — student, investment, and tourist visas are much “better” options. Shuhan Lin (personal communication, October 2020) finds it puzzling that Chinese emigration and investment in Taiwan are allowed, but asylum seekers are shut out: “*We understand there is a national security concern, and we don’t mind a stricter examination of the Chinese people. An actual asylum law would allow for a proper vetting system, based on which the authorities could interview them and know who they are, without that, they just live in our society illegally*.” As there is no legal standard, there is no clear review mechanism for the current political asylum applications coming to Taiwan, and this may also lead to national security loopholes. The asylum system in South Korea could provide a stepping stone to the drafting of a Taiwanese asylum law based on a similar concept.

### Overwhelming the Asylum System

Another argument against including PRC citizens in the law is the fear of the so-called “floodgates” — people pouring out of China and Hong Kong (or even other places) in large numbers, which could overwhelm Taiwan and its new asylum system. However, having systems to manage such scenarios is better than not having them, since a large influx of people can happen whether one plans for it or not. Moreover, the asylum policy could be comprised of two systems, where persons from the PRC, Tibet, Hong Kong, and Macau (group 1 in this research) follow a different mechanism than persons from other countries (group 2 in this research).

The above-mentioned are the three most commonly repeated (valid) arguments against adopting an asylum law. That said, there is not much discussion about asylum in Taiwanese society, nor are there any national sample surveys on people’s attitudes towards refugees and an asylum policy. The reason for this may be the fact that there are not that many asylum seekers and thus people are not very concerned with this topic. And the government has (wisely) kept it that way. That is why many experts in Taiwan regard the year 2016, when the asylum law was discussed in Parliament, as a (welcome) anomaly, and believe the momentum is now lost (CHEN, personal communication, October 2020).

The Hong Kong issue has, in a way, revived the discussion about asylum in Taiwan. Many activists in the country criticize Taiwan’s ruling party for having claimed to support Hong Kong before the election in January 2020, but then refusing to establish a proper asylum law on the pretext of (1) not wanting to provoke Beijing and (2) preventing CCP spies from entering Taiwan as refugees. The DPP’s refusal to enact the asylum law may in effect have been an attempt to avoid triggering an economic backlash from the CCP, which would have angered Taiwan’s bourgeoisie with close commercial ties with China. In response to calls for the creation of an asylum law that would allow Hong Kong protesters to seek refuge in Taiwan, Premier Su Tseng-chang said they could be accepted also under existing laws. We have seen in the previous chapter that dozens of these people remain unsettled and are dependent on the help of civil society groups. The government ended up setting up a Taiwan–Hong Kong Service and Exchange Office to avoid being criticized even further (CHIU, personal communication, October 2020). China’s Taiwan Affairs Office responded with a “warning [to] the Democratic Progressive Party to not … stir things up” (Wei 2020).

## Conclusion

Taiwan is considered to be one of the most progressive countries in Asia, but it has no asylum law, with the “China Factor” often cited as the reason for this. It is ironic, since the existence of the ROC is the outcome of a government led by refugees — people, rich and poor, who ran away from the Mainland between 1949 and 1953 in huge numbers, constituting at that time around 25% of the island’s population.

Compared to UN members, Taiwan is on its own when it comes to the asylum issue. Nevertheless, as a modern and civilized society, which is concerned about human rights, Taiwan needs to address this issue as soon as possible, starting with a more active social dialogue on issues related to the presence of third-country nationals, including refugees. One of the neighbors, South Korea, actually provides a very relevant two-system example for Taiwan, since by the constitution, North Koreans cannot be called foreigners. Taiwan should be smart in its round two of the asylum law drafting and deal with group 1 (persons from the PRC, Tibet, Hong Kong, and Macau) and group 2 (persons from other countries) separately, thus formulating an asylum law based on two systems with very strict standards. However, additional comparative research is needed on this point. Research in this could inform the development of an effective Taiwanese system similar to the South Korean one.

This article has shown that there are several groups of people that are in need of protection in Taiwan and the government is not able to respond effectively in the absence of an asylum law, even though it is bound by the non-refoulment principle (by having domestically adopted the ICCPR).

Moreover, international human rights conventions, some of which Taiwan has ratified and adopted as national law, do not mandate countries to act only once a problem arises. On the contrary, they require the creation of an appropriate legislative framework, whether refugees come or not.

Taiwan also needs to have a ”toolbox” ready for the future when something serious happens and people start to pour in, as we saw in the past when so many people were escaping Vietnam by boat, or more recently people from Hong Kong escaping persecution (although with the pandemic, there has been a halt to their emigration). There are, of course, many more scenarios, and when people start arriving *en masse,* civil servants will not know what to do, and will also need guidance.

While the process of drafting a new asylum law will surely take years, the government of Taiwan could amend relevant clauses in the AGRPTAMA, the LRRHKMC, and the Immigration Act. In addition, it should provide training in relevant issues regarding the *non-refoulment* principle for immigration personnel, front-line law enforcers, and legal professionals and provide at minimum a temporary stay arrangement, which would include respect for the principle of *non-refoulment*. If Taiwan is not willing to become a host country for asylum seekers, then it should facilitate their transfer to third countries with genuine protection mechanisms for refugees. This may seem politically difficult, but in the past, it was possible (when Chinese asylum seekers in Taiwan were sent to the United States or when a North Korean asylum seeker was sent to South Korea). As for Hong Kong protesters facing persecution, Taiwan could offer (unilaterally) 1-year work and travel visas to Hong Kong people under the age of thirty, as is the case with fifteen other countries.

## **Appendix.** International mechanisms regulating refugee protection and Taiwan’s involvement (if any)

**Table:**

1. According to the **Universal Declaration of Human Rights** (1948), adopted by 58 member states which then constituted the UNGA, “*everyone has the right to seek and to enjoy in other countries asylum from persecution*” (UN 1948). On behalf of the ROC, the then-representative of China, it was Chinese diplomat and vice chairman of the Human Rights Commission Peng Chun Chang who attended the conference and is considered one of the most important authors of the declaration (besides the American political figure Eleanor Roosevelt and French legal expert René Cassin). Chang deserves the credit for the universality and religious ecumenism that are now regarded as the declaration's defining features (ROTH 2018).	
**2. The Geneva Convention** (The Convention Relating to the Status of Refugees, 1951) is the centerpiece of the legal definition of the right to asylum. This UN treaty commits its parties to granting asylum to persons with legitimate claims, which include asylum seekers who face political, religious, or ethnic persecution in their home countries. Even 69 years after its creation, it is still the best available tool to protect those most in need.	
**3. The New York Protocol** (The Protocol Relating to the Status of Refugees, 1967) lifted the time and space limitations of the Geneva Convention, since initially, it was applicable only to events before 1951, and mostly in Europe. The PRC, then the new representative of China, became a state party to the Geneva Convention and its later Protocol in 1982 (although the signature does not apply in Hong Kong and Macau), while the ROC is not a signatory to either of these.	
**4. The Convention Relating to the Status of Stateless Persons** (1954) sets the legal framework for the standard treatment of stateless persons. It was adopted to cover, *inter alia*, those stateless persons who are not refugees and thus are not covered by the 1951 Convention relating to the Status of Refugees or its later Protocol (UNHCR 2001). Neither the ROC nor PRC signed the convention (although the convention applies to Hong Kong, a former British colony).	
5. In **the Convention on the Reduction of Statelessness** (1961), originally intended as a Protocol to the above-mentioned Convention, states agreed to reduce the incidence of statelessness (BIANCHINI 2009). Neither the ROC nor PRC are signatories to this convention.	
**6. The UN Convention on the Rights of the Child** (1989) deals with refugees below 18 and with other provisions, such as family reunification. The PRC is one of the original signatories, while the ROC implemented the convention in the country based on the *Implementation Act of the Convention on the Rights of the Child* in 2014 (MINISTRY OF EDUCATION 2019).	
**7. The International Covenant on Civil and Political Rights** (1976) is a multilateral treaty adopted by United Nations General Assembly in 1996 and in force from 1976. Based on the covenant, its parties must respect civil and political rights, such as the rights to life, freedom of religion, or speech. Under the *non-refoulement* obligation, even without a refugee bill, states are required not to expel any person to another territory if this would result in exposing them to the danger of arbitrary deprivation of life, torture, or other cruel, inhuman or degrading treatment or punishment. Taiwan accepted the covenant by incorporating it into its domestic legal system in 2017.	

## Footnotes

1 In this article, “China” refers to the People's Republic of China (PRC) and “Taiwan” to the Republic of China (ROC). Since the revolution in 1949, both the PRC and ROC claim to be the "official" China. Internationally, the PRC is widely recognized as the official China. The government of the PRC considers Taiwan a "renegade province".

2 *The International Covenant on Civil and Political Rights* (ICCPR) is a multilateral treaty adopted by United Nations General Assembly in December 1996 and in force from 1976. Based on the covenant, its parties must respect civil and political rights, such as the rights to life, freedom of religion or speech.

3 *The International Covenant on Economic, Social and Cultural Rights* (ICESCR) commits its parties to work toward granting people economic, social and cultural rights, including labor rights, the right to health, education and adequate standard of living.

4 The principle is considered “patriarchal“ because (1) before the revision of the Nationality Law in 1999, only descendants of male ROC passport holders could apply for naturalization; and because (2) before naturalization without attachment to a Taiwanese national (usually a Taiwanese man), any married immigrant – mostly women from China and Southeast Asia –has to leave when the temporary residency permit expires.

5 The difference between migrant workers and skilled workers is that migrant workers are considered less skilled and they have to leave Taiwan after their job contract expires, whereas “skilled workers” can apply for permanent resident status after living in Taiwan for five years.

6 The Tibetans in India first lived in refugee camps and worked as road builders. Later on, the Dalai Lama asked then-Prime Minister Jawaharlal Nehru to provide them with some land where they could settle together and keep their culture and religion. Karnataka in South India became their new home, where many temples similar to those in Tibet were built. The capital is Dharamshala in the north, and there is a huge settlement in Delhi, too. The exiled Tibetans survive by setting up Tibetan sweater markets in winter in various Indian cities (based on the so-called moral economy principle, meaning they sell with a fixed price and rotate the location in the market). The reason many Tibetans escape to India is that they want to see the Dalai Lama and learn about their culture and religion (which is limited in Tibet by the Chinese authorities). Since 2008, however, only very few manage to go to India, since upon Chinese initiative the border has been sealed.

7 This council was created when the ROC was established and was supposed to take care of Mongolians and Tibetans in the whole territory of China; in reality, its jurisdiction was after 1949 limited to the territory of Taiwan, and thus it has almost no function. Many regarded it as a waste of tax-payer money. Its tasks were shifted to the Ministry of Culture and a small part also to the Mainland Affairs Council. Naturalization is handled by the Ministry of Interior.

8 The principle of *non-refoulement* requires states not to expel any person to another territory if this would result in exposing them to the danger of arbitrary deprivation of life, torture or other cruel, inhuman or degrading treatment or punishment.

9 The movement in Turkey has been met with political opposition, largely because it is a religiously motivated group in a state that has had to struggle to remain secular. The movement spread worldwide establishing schools, hospitals, and media outlets, without any formal hierarchy, or conditions of membership. Gülen himself denies being the leader of anything, and many people in the movement prefer to call it the Hizmet movement (*hizmet* being the Turkish word for selfless service).

10 Even if the child is registered with the respective *de facto* embassy, citizenship is only acquired after following the necessary procedures in the respective country, and so the child remains technically stateless in Taiwan, but with legal residency.

11 Only children with a defined nationality can enter the Taiwanese education system. Even if a stateless child obtains an ARC and enters the schooling system, a graduation certificate will not be awarded as they can only enroll in the form of “auditing”. This has blocked the path to higher education for stateless children, which greatly hinders their future.
